# Cognitive Flexibility and Inhibition Deficits in HIV and Cocaine Dependence: Evidence from Stroop and Trail Making Tests

**DOI:** 10.3390/v18010122

**Published:** 2026-01-16

**Authors:** Sarah E. Nigro, Minjie Wu, Betty Jo Salmeron, Sharmin Islam-Souleimanova, Eve Lauer, Anthony C. Juliano, Alinda R. Lord, Atash Sabet, Lisa H. Lu, T. Celeste Napier, Audrey L. French, Howard J. Aizenstein, Yihong Yang, Shaolin Yang

**Affiliations:** 1 Department of Psychiatry, University of Illinois at Chicago, Chicago, IL 60612, USA; nigro@neurology.wisc.edu (S.E.N.); atash_sabet@yahoo.com (A.S.); 2 Department of Psychiatry, University of Pittsburgh, Pittsburgh, PA 15213, USA; miw75@pitt.edu (M.W.); aizen@pitt.edu (H.J.A.); 3 Office of the Clinical Director, Intramural Research Program, National Institute on Drug Abuse, National Institutes of Health, Baltimore, MD 21224, USA; bsalmeron@intra.nida.nih.gov; 4 Neuroimaging Research Branch, Intramural Research Program, National Institute on Drug Abuse, National Institutes of Health, Baltimore, MD 21224, USA; sharminis2@gmail.com (S.I.-S.); ethao1@jh.edu (E.L.); yihongyang@intra.nida.nih.gov (Y.Y.); 5 Department of Public Health, Johns Hopkins Bloomberg School of Public Health, Baltimore, MD 21205, USA; 6 Department of Psychiatry, University of Vermont, Burlington, VT 05405, USA; anthony.juliano@uvm.edu; 7 Department of Psychology, Roosevelt University, Chicago, IL 60605, USA; 8 Department of Psychiatry, Rush University Medical Center, Chicago, IL 60612, USA; celeste_napier@rush.edu; 9 Department of Medicine, CORE Center/Stroger Hospital of Cook County, Chicago, IL 60612, USA; afrench@cookcountyhhs.org

**Keywords:** HIV/AIDS, drug use disorder, executive functions, quality of life

## Abstract

Objective: To better define potential executive function difficulties in individuals living with HIV but not clinically identified as having HAND, with and without mild to moderate cocaine dependence (CD), our cross-sectional study examined executive function performance on the Stroop Color-Word Test (Stroop) and the Trail Making Test (TMT) in four groups stratified by HIV and CD status. Method: We recruited 101 participants (26 HIV+/CD+; 18 HIV+/CD−; 30 HIV−/CD+; 27 HIV−/CD−). We utilized a 2 (HIV: yes/no) × 2 (Cocaine: yes/no) MANCOVA while controlling for age and premorbid intelligence on the Stroop trials (i.e., color-naming, word-reading, interference), and TMT-A and TMT-B z-scores, number of errors, and the B/A ratio. Results: HIV was associated with significantly slower performance on the Stroop Interference (*p* = 0.012, η^2^ = 0.064). CD showed a trend towards slower performance on interference trials (*p* = 0.061, η^2^ = 0.037) and was associated with significantly more errors on the Stroop Word-Reading (*p* = 0.028, η^2^ = 0.050) and Interference trials (*p* = 0.046, η^2^ = 0.041), suggestive of difficulties with inhibitory control and written language processing. There were no significant HIV × Cocaine interactions. Conclusions: Our results suggest HIV without clinically identified cognitive impairment and CD are associated with distinct and potentially overlapping executive functioning deficits, particularly for measures of inhibitory control. Notably, CD showed trend-level slowing on Stroop Interference performance, suggesting partial overlap with HIV effects. Clarifying the specific cognitive processes impacted by HIV and CD can help guide tailored interventions to improve functional outcomes in these populations.

## 1. Introduction

Individuals living with human immunodeficiency virus (HIV) commonly demonstrate a neuropsychological profile characterized by prominent disturbances in executive functioning [1,2,3,4,5]. In addition to the cognitive impairment associated with HIV, there is a high rate of substance use disorders within this population, making it difficult to determine if the notable cognitive disturbances are related to the HIV disease process and/or substance use. Stimulants, such as cocaine, are among the most frequently used substances in the HIV population [6,7,8]. Active, ongoing cocaine use accelerates HIV progression [9], rendering cocaine use especially dangerous in this group. Executive functioning is one of the cognitive domains most impacted by both HIV and cocaine use [8,10,11].

Although many studies have examined executive functioning in individuals with HIV and in cocaine dependence (CD) populations, few have explored the specific underlying cognitive processes affecting performance on executive functioning tasks. Specifically, there is a lack of direct comparisons between different executive functioning measures in individuals living with HIV and comorbid cocaine use. A recent systematic review and meta-analysis [12] highlighted considerable differences within the conceptualization and measurement of executive functioning within the HIV population. While there is a consensus that executive functioning impairments exist in both HIV and cocaine use populations, it remains unclear which aspects are uniquely impacted by each condition, especially when they co-occur.

Our study aimed to address this gap by examining two executive functioning measures assessing distinct aspects of executive functioning. One of the neuropsychological tests is the Stroop Color-Word Test (Stroop) [8,13]. Although versions of the Stroop vary, they all include an Interference trial, in which the individual is instructed to name the ink color of the words printed and not read the word (i.e., the word RED is printed in green ink, so the correct response would be “green”). The Interference trial is where the “Stroop effect” is observed [14]. The Stroop effect refers to the delay in response time observed when incongruent words interfered with naming the ink’s color. The Stroop task provides insight into an individual’s processing speed, visual scanning, inhibition, and sustained attention abilities. Because appropriate, demographically representative norms for the Comalli–Kaplan Stroop are limited for Black/African American adults, raw Stroop scores are used to avoid misclassification of impairment in this population.

The literature on Stroop performance in individuals living with HIV has shown mixed results. Some studies report no significant differences on the Interference trial between individuals with and without HIV infection [1,15,16,17]. Others have found individuals living with HIV perform significantly worse on the Interference trial than individuals without HIV infection [3,18,19]. Notably, comorbidity with substance use disorders has not been handled consistently in these studies. In contrast, the literature on cocaine use (whether a related diagnosis was assessed or not) has more consistency in demonstrating difficulties on the Interference trial and a higher number of response errors [10,20,21].

Another commonly used executive functioning measure is the Trail Making Test (TMT). The TMT has two parts (i.e., TMT-A and TMT-B). TMT-A primarily assesses processing speed and visual scanning, whereas TMT-B primarily measures set-shifting/cognitive flexibility. A key difference between TMT and Stroop is TMT’s reliance on fine-motor skills. Findings on TMT performance in the HIV population have also been mixed. Some studies show similar performances on TMT-B between individuals with and without HIV infection [22,23], whereas others have found individuals living with HIV perform worse than individuals without HIV infection [18,24,25]. Multiple studies have found no difference in the TMT when comparing performances between individuals with and without cocaine abuse or dependence [26,27]. Of note, the studies mentioned above did not control for motor speed. Since TMT requires an individual to draw lines as quickly as possible, an individual’s fine motor abilities can impact task performance [28]. As a result, researchers have suggested using derived scores (i.e., B/A ratio) to minimize the effects of motor speed and isolate executive functioning deficits [29,30].

Another significant factor impacting the consistency of findings in the literature is the lack of control for polysubstance use, making it extremely challenging to determine which substance(s) affect an individual’s performance. It is critical to understand the effects of cocaine, in isolation of other substances, to provide clarity as to the specific impact cocaine has on cognitive functioning and the importance of addressing it in the clinic. The high percentage of cocaine use within the HIV population, particularly within minority groups [31], results in a great need to understand the effects of cocaine within the HIV population. Lastly, there is a need for clarity in the literature given the inconsistencies in findings which could be due to the inability to determine which substance is causing what deficits or which substance may interact poorly with HIV with respect to executive function. Therefore, there is a pressing need to examine the effects of cocaine use [21] independent of other substances, which our study explicitly sought to address.

In the present study, we minimized potential confounding variables through strict exclusion and inclusion criteria. Our goal was to examine the effects of well-controlled, asymptomatic HIV infection and cocaine use on specific aspects of executive functions. By focusing on asymptomatic HIV infections, we aimed to capture the impact of CD on those who seem to be doing well with their HIV infection in order to determine if CD may impact cognitive functioning in otherwise clinically stable individuals with HIV. We required individuals to meet DSM-IV criteria for CD but also restricted our sample to individuals who could present for the study with a negative urine toxicology screen. This served two purposes. First, studying only those who can present with a negative urine toxicology screen isolates differences to the chronic effects of having CD rather than the acute effects of intoxication. Second, those whose CD is severe enough that they are unable to abstain long enough to produce a negative urine screen are very likely to need clinical intervention directed towards their CD, while it is less likely to come to clinical attention and less clear how important it is to intervene with those who can abstain for short periods of time. Because this requirement necessarily restricted enrollment to individuals able to abstain long enough to produce a negative toxicology screen, the resulting cocaine-dependent cohort represents a relatively higher-functioning group with generally mild to moderate severity. As such, the findings may not generalize to individuals with more severe CD or those with highly active cocaine use. To examine the individual and combined effects of both HIV and CD, our sample consisted of four groups; individuals living with HIV and CD (HIV+/CD+), individuals living with HIV without CD (HIV+/CD−), individuals without HIV with CD (HIV-/CD+), and individuals with neither HIV nor CD (HIV−/CD−).

Based on prior research, we hypothesized that individuals living with HIV (HIV+/CD+ and HIV+/CD−) would exhibit poorer performance (i.e., take significantly longer) on both the Stroop Interference trial and TMT-B. We also predicted individuals with CD but without HIV (HIV−/CD+) would perform worse on the Stroop Interference (i.e., longer completion time), but perform comparably to controls on TMT-B. Lastly, based on previous research, we predicted a significant interaction between HIV and CD on the Stroop Interference and TMT-B, with the HIV+/CD+ group displaying the most substantial impairments relative to the other groups.

## 2. Materials and Methods

### 2.1. Design

This study was a cross-sectional, 2 (HIV: yes/no) × 2 (CD: yes/no) factorial design comparing performance on the Stroop Test and the TMT across four groups (i.e., HIV+/CD+, HIV+/CD−, HIV−/CD+, and HIV−/CD−).

### 2.2. Inclusion/Exclusion Criteria

Participants were required to be between 21 and 55 years of age, fluent in English, have transportation to and from the study site, and be capable of providing informed consent (Table 1).

All substance use diagnoses in this study, including CD, were based on the Structured Clinical Interview for DSM-IV-TR (SCID-IV-TR). At the time of data collection, DSM-IV diagnostic criteria distinguished between substance abuse and dependence, with dependence representing a more severe and chronic pattern of use characterized by tolerance, withdrawal, and compulsive drug-seeking behaviors. Although the DSM-5 has since consolidated these into a single “substance use disorder” category with severity specifiers, the use of DSM-IV criteria was appropriate for this study’s aims and timeline. As such, the term “cocaine dependence” (CD) is retained throughout this manuscript to reflect the specific diagnostic criteria applied during participant enrollment and data collection.

Exclusion criteria included: a positive urine drug screen, current or lifetime DSM-IV diagnosis of schizophrenia or other psychotic disorders, a history or current diagnosis of neurodevelopmental, neurocognitive, or mood disorder other than major depression and generalized anxiety disorder (e.g., ADHD, Intellectual Disability), a history of head injury with a loss of consciousness exceeding 30 min, or uncorrected vision problems. Individuals in the HIV-negative groups were required to provide medical documentation verifying a negative HIV test within 6 months of enrollment. Those unable to do so completed a rapid oral HIV test. HIV-associated neurocognitive disorder (HAND) was excluded using documentation review and participant history, rather than formal diagnostic criteria, as this was not a clinical evaluation. Medical records were reviewed for any prior HAND diagnosis or CNS opportunistic infections, and participants were asked whether they had ever been told they had HAND. Because this was not a clinical evaluation, we did not apply Frascati criteria; instead, individuals with known or suspected HAND based on these sources were excluded. Additional exclusion criteria for individuals within the CD− groups were current or lifetime DSM-IV diagnosis of abuse or dependence on cocaine, opioids, or methamphetamine; DSM-IV diagnosis of dependence on any other illegal or prescription drug within the past 5 years; or DSM-IV diagnosis of dependence on alcohol within the past year.

All individuals in the CD+ groups were required to meet DSM-IV criteria for a CD diagnosis within the past two years but be able to produce a negative urine on their test day. Exclusion criteria were a current or lifetime DSM-IV diagnosis of abuse or dependence on opioids or methamphetamine; DSM-IV diagnosis of dependence on any other illegal or prescription drug within the past five years; and DSM-IV diagnosis of alcohol dependence within the past year. Substance use criteria aligned with existing literature [8,32,33].

Individuals living with HIV were required to be adherent to HIV medications for at least the past three months, confirmed via the Medication Adherence Self-Report Inventory (MASRI), have a CD4 count greater than 200, and have an undetectable HIV RNA viral load (<50 copies/mL). Individuals with a history of HAND or central nervous system opportunistic infections (e.g., progressive multifocal leukoencephalopathy, toxoplasmosis, cryptococcal meningitis, or CNS lymphoma) were excluded. Excluding HAND allowed us to focus on those whose cognitive issues had not yet become a clinically recognized problem. The specific criteria for those living with HIV were set in place to ensure that all individuals with HIV were asymptomatic, according to the Centers for Disease Control and Prevention (CDC) and the World Health Organization (WHO) HIV disease severity staging system [34]. Notably, we relied on self-report and medical records to assess this criterion, not direct neuropsychological testing. Participants were not required to demonstrate intact cognition but did not have symptoms that had come to clinical attention. Our aim with these extensive criteria was to focus on relatively functional individuals living with HIV and CD for whom the importance of addressing the CD is less obvious and for whom the cognitive impact of each disorder is less clear. The HIV specific criteria are consistent with previous studies that aimed at minimizing confounding factors potentially related to HAND [35].

### 2.3. Procedure

This study received approval from the University of Illinois at Chicago Institutional Review Board. Participants were recruited from local clinics within Chicago’s 560-acre Illinois Medical District (i.e., the Chicago Developmental Center for AIDS Research, the University of Illinois at Chicago Infectious Disease Clinic, and the Ruth M. Rothstein CORE Center). Interested individuals contacted the study staff and completed a phone screen to assess the initial eligibility. Eligible participants who agreed to abstain from drug and alcohol use long enough to produce a negative urine drug screen (advised for at least 48 hours) were scheduled for an in-person visit. At the visit, participants completed informed consent procedures followed by a urine toxicology test (screening for amphetamines, benzodiazepines, barbiturates, cocaine, opioids, and THC) and an alcohol breathalyzer test. Visits were rescheduled if either test was positive. If screening was passed, participants proceeded with study procedures.

### 2.4. Measures

#### 2.4.1. Screening Measures

Participants completed a demographic questionnaire and the SCID-IV-TR [36] to assess for any developmental, mood, or psychiatric diagnoses relevant to eligibility and potential confounding factors (e.g., depression, ADHD, intellectual disability). Participants also completed two self-report measures related to mood symptoms that could impact performance on the neuropsychological measures, including the 10-item Perceived Stress Scale (PSS-10; [37]) and the Center for Epidemiologic Studies Depression Scale (CES-D; [38]).

#### 2.4.2. Substance Use

Module E of the SCID-IV-TR was explicitly utilized to determine specific current and lifetime substance abuse and dependence. Participants also completed the Kreek–McHugh–Schluger–Kellogg Scale (KMSK) to assess current (i.e., within the past month) and historical substance use (i.e., in their lifetime). The KMSK provides critical values, calculated from the participants’ frequency (i.e., number of times used in the past month and lifetime), amount of substance use or amount of money spent daily, and duration of use for each specific substance [39].

#### 2.4.3. Medical Record Review

All participants provided a release of information for medical records related to our study aims. For participants living with HIV, HIV variables including date of HIV diagnosis, most recent (within the past six months) CD4 count, HIV RNA viral load (copies/mL), and current list of medications were all recorded. Participants living with HIV also completed the MASRI. For participants without HIV infection, if they could not confirm their HIV status within the past six months with medical documentation, they were given the OraQuick^©^ rapid HIV test (OraSure Technologies, Inc., Bethlehem, PA, USA).

#### 2.4.4. Neurocognitive Functioning

Trained graduate research assistants administered a brief neuropsychological test battery (Supplemental Table S1). Two research assistants double scored all measures. We administered the Wide Range Achievement Test, Fourth Edition: Word Reading subtest (WRAT-4: Word Reading) [40], a well-validated measure of premorbid cognitive functioning [41]. Of note, participants were not excluded based on low performance scores on any of the neuropsychological test battery; the battery was solely for assessment purposes.

To assess executive functioning, we utilized both the Stroop Color-Word Test (Stroop) and TMT. The Stroop Color-Word Test, Comalli–Kaplan version [42], consists of three trials. The Color-Naming trial (Trial 1) and the Word-Reading trial (Trial 2) are primarily for the attention and processing speed measures. The Interference trial (Trial 3) is mainly for a measure of response inhibition and cognitive flexibility. Completion time for each trial was recorded in seconds, with a discontinue rule of four minutes on each of the three trials. The number of errors made on each of the trials was also recorded. Errors were defined as errors participants did not self-correct. The Stroop’s existing norms have been found to over pathologize Black/African Americans [35], which is most of our sample. Because the Comalli–Kaplan version of the Stroop Color-Word Test does not have up-to-date published norms representative of this study’s sample, we utilized the raw scores. However, we acknowledge that use of raw scores limits cross-study comparability, and this is noted as a methodological limitation.

The TMT has two parts, i.e., TMT-A and TMT-B. TMT-A consists of 25 numbered circles in which a person draws a line connecting each of the numbered circles in numerical order. TMT-B consists of both numbers (1–13) and letters (A–L) in which a person is to draw a line connecting numbers and letters in order switching from a number to a letter (i.e., 1-A-2-B-3-C). We recorded completion time (in seconds) and number of errors made. An additional derived ratio score (B/A) was computed to measure executive functioning more directly while controlling for the impact of visual-motor speed [29,30]. A higher TMT B/A ratio suggests reduced cognitive flexibility or set-shifting capability. The TMT-A primarily measures visual-motor processing speed, and TMT-B primarily measures cognitive switching [43,44]. We utilized the Heaton norms, which took sex, age, race/ethnicity, and education level into consideration to convert raw scores to demographically corrected z-scores [45]. To supplement the demographically corrected z-scores, raw completion times and error counts for TMT-A and TMT-B are provided in Supplemental Table S2.

### 2.5. Statistical Analysis

Data were analyzed using the Statistical Package for the Social Sciences (SPSS), version 25.0 (IBM Corp., Armonk, NY, USA). For each set of analyses, all data were examined for distributional properties. A 2 (HIV+/HIV−) × 2 (CD+/CD−) multivariate analyses of covariance (MANCOVA) with premorbid IQ and age as our covariates was utilized to examine differences in completion time and number of errors on both the Stroop and TMT outcome variables.

Age was included as a covariate as our sample significantly differed in age between groups; additionally, we included premorbid IQ in the model, this is because although premorbid IQ did not significantly differ between groups, it was significantly correlated with our dependent variables.

To further examine if the Stroop and TMT measures assessed similar cognitive processes, we performed correlation analyses between the Stroop and TMT measures. To control for multiple comparisons, we utilized Tukey’s HSD correction to determine the significance of these omnibus tests.

Chi-square tests were used to determine if there were significant differences between categorical variables, and two-way analysis of variance (ANOVA) was used for all the continuous variables. For individuals living with HIV, *t*-tests were used to determine if there were significant differences between those with and without CD in the mean CD4 count, HIV RNA viral load (copies/mL), and years since HIV diagnosis. For individuals with CD, *t*-tests were used to determine if there were significant differences between those with and without HIV in age of first use and days since last use. There was a significant between-group difference in age of first use (*p* = 0.02); however, age of first use was not significantly correlated with any of our outcome measures (*p* > 0.05). A chi-square test was used to identify any differences in CD diagnosis, and *t*-tests for all KMSK scores were used to determine if there were any significant differences in all other substance use between the CD groups (Table 2). There were no significant differences in these CD variables.

## 3. Results

### 3.1. Participants

**Figure 1:**
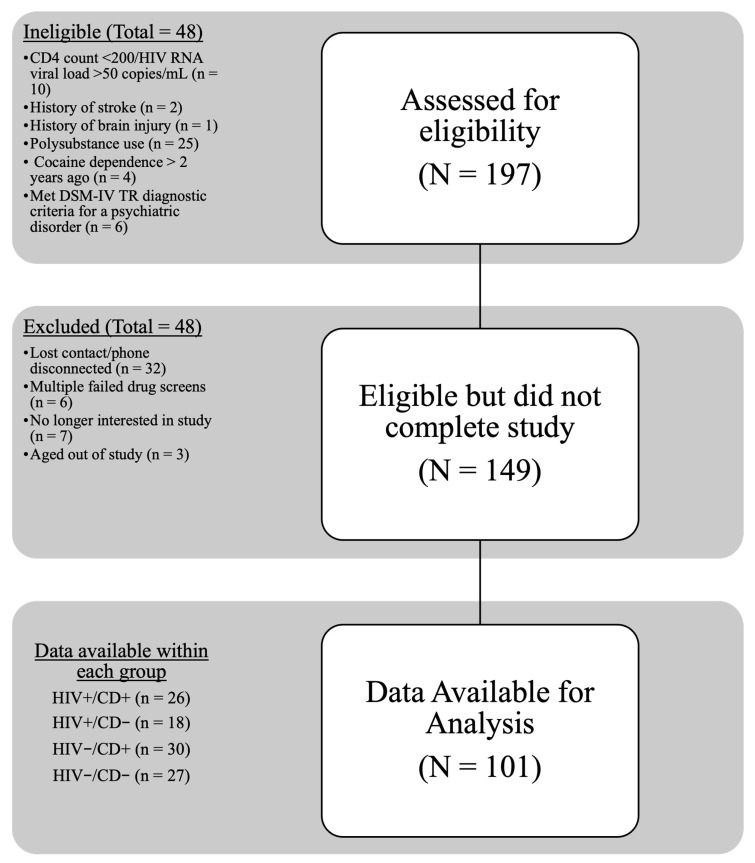
Study Flow Diagram.

Demographic and clinical characteristics of the sample are detailed in Table 1. Of the 197 participants assessed for eligibility, 101 participants completed the study (Figure 1), including 26 HIV+/CD+, 18 HIV+/CD−, 30 HIV−/CD+, and 27 HIV−/CD− individuals. The overall sample consisted of 56 males and 45 females. Most participants identified as Black/African American (75.2%), with the remainder identifying as Hispanic/Other (15.8%) or White/Non-Hispanic (8.9%). The mean level of education was 13.43 years (*SD* = 2.09), and the participant age ranged from 22 to 55 years old (*M* = 42.12, *SD* = 9.42). There was a significant difference in age between groups (*p* = 0.002), with the HIV−/CD− group significantly younger than the HIV+/CD+ group (*p* = 0.001). Premorbid IQ (WRAT-4: Word Reading) and years of formal education were comparable across groups.

There were no significant differences in either peak or recent cocaine dependence severity between the two CD groups. Notably, although all cocaine-dependent participants met diagnostic criteria within the past two years, the relatively low KMSK current scores (mean approximately 4–5 out of 26) indicated mild to moderate current severity, reflecting relatively controlled cocaine use at the time of testing. Mild to moderate severity may indicate our CD participants were generally higher-functioning and may not represent individuals with severe or ongoing CD. Among participants living with HIV, there were no significant differences in years since HIV diagnosis, CD4 lymphocyte count, or HIV RNA viral load.

### 3.2. Neurocognitive Functioning

**Figure 2:**
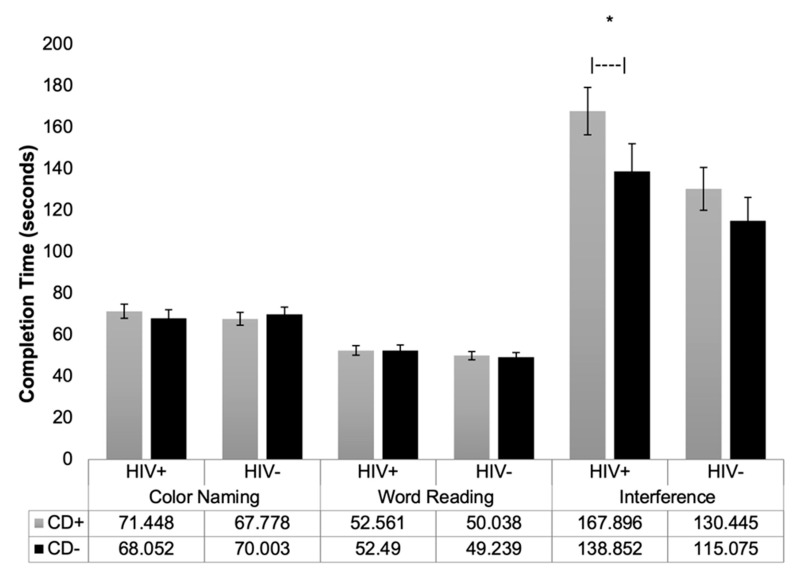
Mean Completion Time on Stroop Interference Trial by HIV and CD Status. The mean completion time (in seconds) for each of the three Stroop trials is shown for each group (*N* = 101). Error bars indicate standard errors. Horizontal brackets denote main effects for HIV status and CD status. Note. * *p* = 0.012 for main effect of HIV status. Patterns shown reflect the results across all four groups (HIV+/CD+, HIV+/CD−, HIV−/CD+, HIV−/CD−), consistent with omnibus and pairwise analyses.

**Figure 3:**
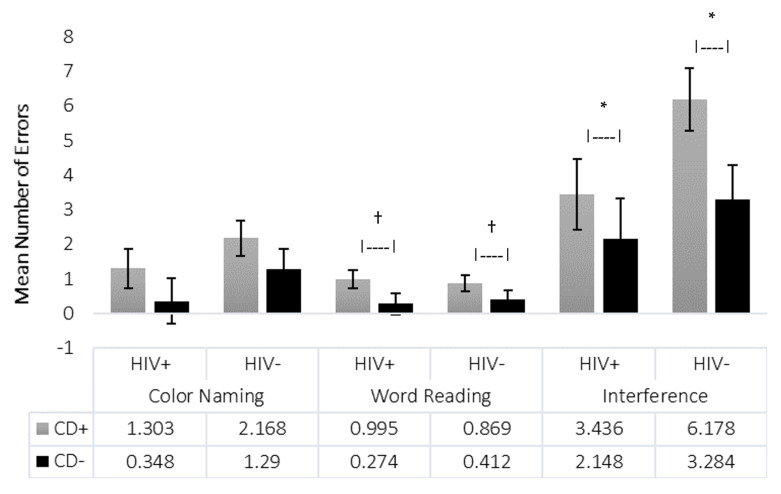
Stroop Number of Errors. The mean number of errors for each of the three Stroop trials is shown for each group (*N* = 101). Standard errors are represented in the figure by the error bars attached to each column. Horizontal brackets denote main effects for HIV status and CD status. Note. † *p* = 0.028 for main effect of CD status. * *p* = 0.046 for main effect of CD status. Patterns shown reflect the results across all four groups (HIV+/CD+, HIV+/CD−, HIV−/CD+, HIV−/CD−), consistent with omnibus and pairwise analyses.

**Figure 4:**
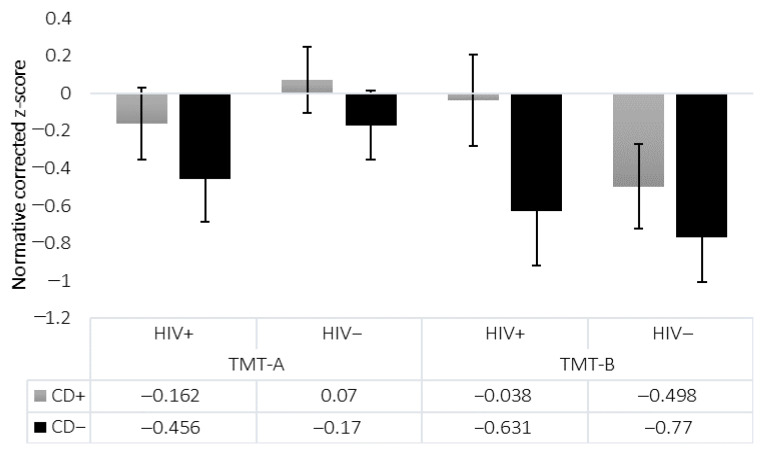
Trail Making Test (TMT) Normative Corrected Z-Scores. Shown are the mean normative corrected z-scores for completion time of TMT-A and TMT-B (*N* = 101). Standard errors are represented in the figure by the error bars attached to each column. There were no significant main or interactive effects. Patterns shown reflect the results across all four groups (HIV+/CD+, HIV+/CD−, HIV−/CD+, HIV−/CD−), consistent with omnibus and pairwise analyses.

**Figure 5:**
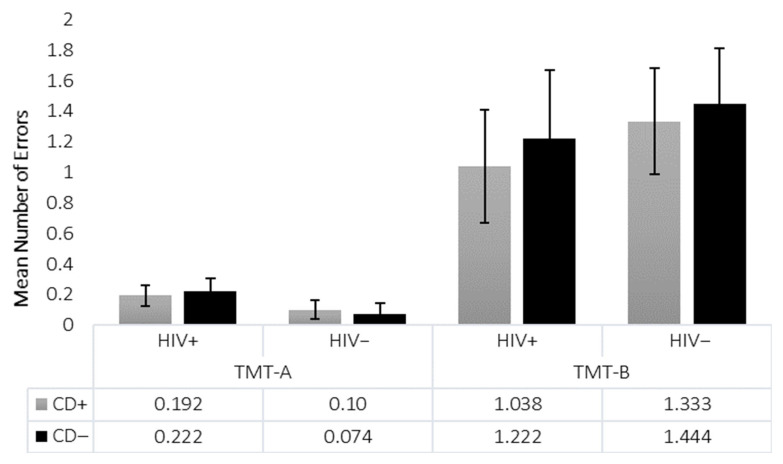
Trail Making Test (TMT) Mean Number of Errors. Shown is the mean number of errors made on TMT-A and TMT-B for each group (*N* = 101). Standard errors are represented in the figure by the error bars attached to each column. No significant differences are found. Patterns shown reflect the results across all four groups (HIV+/CD+, HIV+/CD−, HIV−/CD+, HIV−/CD−), consistent with omnibus and pairwise analyses.

All mean raw scores for the Stroop and z-scores for the TMT are presented in Figure 2, Figure 3, Figure 4 and Figure 5. It is important to note that raw scores and z-scores are not equivalent and must be interpreted independently.

For the Stroop, raw scores were utilized while controlling for age and premorbid IQ. The Comalli–Kaplan version of the Stroop Color-Word Test lacks appropriate normative data for Black/African American populations, who comprised most of our sample; therefore, raw scores were used to avoid over-pathologizing performance. The mean completion times for all three trials are presented in Figure 2.

On the Color-Naming trial, there were no significant main effects of HIV status (*p* = 0.814, η^2^ = 0.001) or CD status (*p* = 0.869, η^2^ < 0.001) on completion time. Similarly, on the Word-Reading trial, neither HIV status (*p* = 0.229, η^2^ = 0.015) nor CD status (*p* = 0.852, η^2^ < 0.001) had a significant effect. However, on the Interference trial, there was a significant main effect of HIV status (*p* = 0.012, η^2^ = 0.064), with slower performance in those living with HIV, and a trend toward significance for CD status (*p* = 0.061, η^2^ = 0.037), with slower performance in the CD+ group. No significant HIV × CD interaction was found for completion time of Color-Naming (*p* = 0.420, η^2^ = 0.007), Word-Reading (*p* = 0. 873, η^2^ < 0.001), or Interference (*p* = 0.551. η^2^ = 0.004) trials. Consistent with the omnibus MANCOVA, pairwise contrasts indicated no significant differences between the HIV−/CD+ and HIV−/CD− groups on any Stroop outcome.

In addition to the completion time, we examined errors made on each trial. Errors were defined as incorrect responses not self-corrected. There were no significant differences in error on the Color-Naming trial. On the Word-Reading trial, there was a significant main effect of CD (*p* = 0.028, η^2^ = 0.050), with CD+ participants making more errors. A similar effect was observed on the Interference trial (*p* = 0.046, η^2^ = 0.041), again with CD+ participants making more errors. HIV status did not affect the number of errors on any of the Stroop trials (all *p* > 0.07; Figure 3).

**Figure 6:**
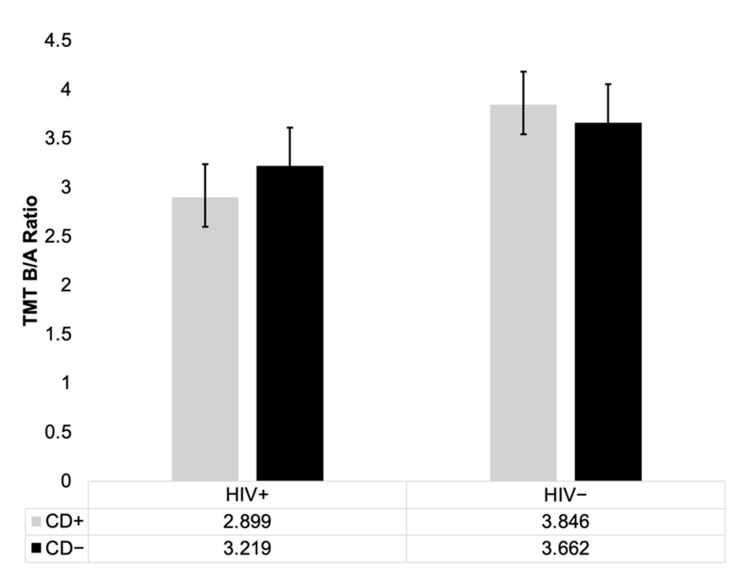
Trail Making Test (TMT) B/A Ratio. Shown is the mean B/A ratio for each group. Standard errors are represented in the figure by the error bars attached to each column (*N* = 101). No significant differences were found. However, a trend-level main effect of HIV (*p* = 0.052) was found. Patterns shown reflect the results across all four groups (HIV+/CD+, HIV+/CD−, HIV−/CD+, HIV−/CD−), consistent with omnibus and pairwise analyses.

For the TMT, demographically corrected z-scores were used [45], and analyses controlled for age and premorbid IQ via MANCOVA. Means and standard errors are shown in Figure 4. On TMT-A, there were no significant main effects of HIV (*p* = 0.183, η^2^ = 0.019), CD (*p* = 0.319, η^2^ = 0.010), or their interaction (*p* = 0.973, η^2^ < 0.001). On TMT-B, similarly, no significant effects of HIV (*p* = 0.180, η^2^ = 0.019), CD (*p* = 0.188, η^2^ = 0.018), or their interaction (*p* = 0.704, η^2^ = 0.002) were observed. Analysis of the TMT-B/A ratio, designed to isolate cognitive flexibility while controlling for motor speed, revealed only a trend-level main effect of HIV (*p* = 0.052, η^2^ = 0.039), and no significant main effect of CD (*p* = 0.844, η^2^ < 0.001), nor HIV × CD interaction effect (*p* = 0.455, η^2^ = 0.006) (Figure 6). No group differences were found in number of errors on either TMT-A (all *p* > 0.40) or TMT-B (all *p* > 0.38). Similarly, no significant differences were observed between HIV−/CD+ and HIV−/CD− groups on either TMT-A or TMT-B (Figure 5).

Finally, Pearson correlations were conducted between TMT and Stroop performance. No significant relationship emerged between TMT-B z-scores and Stroop Interference completion time (*p* = 0.62), suggesting these tasks measure distinct aspects of executive functioning.

## 4. Discussion

This study examined the independent and combined effects of HIV and CD on executive functioning using the Stroop Color-Word Test and the Trail Making Test. Overall, we found that both asymptomatic, treated HIV infection and mild to moderate CD were associated with executive dysfunction, primarily involving inhibitory control. However, no significant HIV × CD interaction effects emerged.

Participants performed similarly across groups on the Color-Naming and Word-Reading Stroop trials. On the Interference trial, however, individuals living with HIV demonstrated significantly slower performance, suggesting impaired response inhibition and cognitive flexibility. This finding remained significant even after accounting for baseline processing speed using difference scores. Cocaine dependent individuals showed trend-level slower performance on Interference trials indicating some similarity to the deficit seen in those living with HIV. The trend-level slowing in Stroop Interference performance for participants with CD suggests potential overlap with HIV-associated impairments in inhibitory control. Given our modest sample size and low power for interaction effects, these trends may reflect meaningful shared deficits that merit exploration in larger, adequately powered studies. No interaction between HIV status and CD was found, in agreement with a published result in women [16].

Regarding accuracy, CD status significantly impacted the number of errors on the Word-Reading and Interference trials, with participants with CD making more errors, consistent with previous findings implicating cocaine use in inhibitory control deficits [21,46,47]. Such deficits have clinical implications including poor treatment compliance, treatment retention, and self-regulation in CD [46,48,49]. In substance use clinics, the Stroop has been used to determine treatment retention and abstinence from substances. Continued CD resulted in decreased inhibition and thus greater difficulty in remaining abstinent from cocaine [47,50], which is consistent with our finding that CD is associated with impaired inhibition. Thus, even mild to moderate severity of CD in this cohort may impair the ability to adhere to HIV treatment regimens, warranting treatment, especially since HIV status itself did not impact error rates.

No significant effects were observed on TMT performance, in line with prior published studies using proper normative data [22]. The selective Stroop impairment reflected in longer completion time on Interference trials among individuals with HIV highlights difficulties in cognitive flexibility in this population, supporting prior findings from other executive functioning measures [12,51,52], while the selective impairment of CD on errors on the Word-Reading and Interference trials points to subtly different executive function deficits in CD possibly related to error monitoring.

The lack of significant HIV × CD interaction effect aligns with some prior studies [8,33,53]. It is possible that our modest sample size limited the power to detect significant interactions. Additionally, variability in time since last cocaine use may also have affected results, as cognitive performance can fluctuate with recency of use [50,54,55]. Therefore, the range within our sample (e.g., CD within past two years) might have impacted our findings. Consistent with their ability to abstain and produce a negative urine toxicology screen, our CD sample also showed mild to moderate current cocaine use severity, which may also explain the lack of interaction. Finally, since we restricted our investigation to individuals with HIV who had not already been identified as having HAND, it may be that CD has less impact on individuals with HIV who have more intact cognition. Importantly, exclusion of individuals with a prior clinical diagnosis of HAND does not imply intact cognition in the HIV-positive sample. Participants were not excluded based on neuropsychological performance, and undiagnosed or subclinical cognitive impairment may have been present at the time of assessment. As such, the observed executive dysfunction may reflect early or subtle cognitive vulnerability rather than the absence of impairment. Future studies incorporating longitudinal designs and neuroimaging methods will be important for clarifying the neural mechanisms underlying these distinct patterns of executive dysfunction.

By examining these two executive functioning measures, we could better understand the underlying cognitive processes associated with their overall performance on executive functioning. Clinically, HIV and CD appear to impact different aspects of executive functioning. Inhibitory control and error monitoring in CD and cognitive flexibility in HIV likely reflect distinct neural network involvement. This distinction between HIV-related cognitive flexibility challenges and cocaine-related inhibitory control difficulties provides a preliminary framework for developing targeted clinical interventions. Impairments in inhibition suggest orbitofrontal dysfunction in CD, while deficits in error monitoring suggest dorsal anterior cingulate cortex dysfunction. Cognitive flexibility deficits in HIV may indicate dorsolateral prefrontal cortex involvement. In contrast, TMT performance may have been less sensitive to these differences because TMT-B places greater demands on visual scanning and motor speed, which were relatively preserved in this sample, reducing the likelihood of detecting subtle executive differences.

Understanding the cognitive differences can guide tailored clinical interventions. For example, individuals with HIV may benefit from clear, structured communication, explicit written instructions, and compensatory cognitive training. Compensatory cognitive training can help these individuals to gain a better understanding of potential impairment with their cognitive difficulties and help them learn critical skills for effectively coping and navigating through their everyday life. Individuals with CD may require interventions targeting inhibitory control and attention to errors, even in cases of mild to moderate severity. By acknowledging these specific impairments in these individuals’ cognitive profiles, we can more adequately address these deficits and help them foster a more positive quality of life.

A notable strength of this study is the use of comprehensive inclusion and exclusion criteria. Participants had to be adherent to their HIV medications within the past three months and have a CD4 count above 200. We verified this information by reviewing their medical records and self-reports on the MASRI. We conscientiously verified participants who were in the HIV+/CD+ group and the HIV−/CD+ group were not actively dependent on any other drugs aside from cocaine. We utilized multiple measures to verify current and historical substance use, including a breathalyzer test, urine drug screening, KMSK, and the SCID-IV-TR substance abuse, Module E. The strict inclusion/exclusion criteria allowed us to examine the effects of cocaine in isolation and significantly reduce the number of confounding variables.

In the present study, we also came across several limitations. First, the sample size within each group was relatively small, which affected the statistical power. However, the small sample size was mainly due to the difficulty in recruiting participants that could abide by the strict eligibility requirements to limit confounding factors. The modest sample size limited statistical power to detect HIV × CD interaction effects, and any trend-level findings should be interpreted with caution. Despite the small sample size, we still found significant differences between the groups, and we interpreted our findings more confidently, given our strict inclusion and exclusion criteria. Second, although multiple measures were used to determine past substance use history, these measures were primarily based on the participants’ self-report, aside from the urine toxicology screen and alcohol breathalyzer test administered at the beginning of each study visit. Any unreported polysubstance dependency could potentially impact the neurocognitive task performance and therefore the interpretation of the findings. Third, we did not collect data on Nadir CD4 count, which has been shown to be associated with cognitive outcomes in those living with HIV. As a result, we cannot determine whether historical immunosuppression contributed to the HIV-related cognitive differences observed in this study. The inclusion of an HIV−/CD+ group is a strength of our study as we were able to understand the effects of CD without the confounding effects of other substances. However, on the other hand, this can also be viewed as a weakness in generalization of our findings to more general populations [8,20,33,56]. Because all participants with HIV were on antiretroviral treatment, which may affect cognitive function, we could not distinguish between the effects of HIV infection and those of treatment on cognition. Future studies should also record the specific antiviral therapies for each participant to determine any possible differences in therapies on cognition. Nevertheless, since treatment adherence is critical for individuals living with HIV to remain healthy, understanding the cognitive challenges in this population remains clinically important. There is also an age difference between the groups, with participants with HIV being younger than HIV-negative participants, which should also be considered in the generalizability of these findings. Additionally, although depressive symptoms and perceived stress were collected using the CES-D and PSS-10 and did not differ significantly across groups, these variables were not included as covariates due to the modest sample size and risk of overfitting. Nonetheless, subtle effects of mood or stress on executive functioning cannot be entirely ruled out. Finally, while we do not view it as a limitation, we do think it is important to again highlight that the focus of this study was on individuals with HIV infection who had not already been identified as having HAND. Cocaine has already been documented to adversely affect those with HAND [57]. Our focus was on elucidating the importance of addressing cocaine use in those with less obvious neurocognitive consequences of HIV infection.

Taken together, these findings should be interpreted as preliminary given the modest sample size and mild level of cocaine dependence in the cohort, and replication in larger and more diverse samples is needed to confirm the separable effects of HIV and cocaine dependence on executive functioning.

## Figures and Tables

**Table 1:** Demographic and Clinical Characteristics.

	HIV+/CD+ (*n* = 26)	HIV+/CD− (*n* = 18)	HIV−/CD+ (*n* = 30)	HIV−/CD− (*n* = 27)	Statistic
Age (SD)	45.54 (6.76)	42.11 (7.10)	41.87 (9.66)	38.15 (11.14)	*F* (3, 97) = 5.22, *p* = 0.002
Years of Education (SD)	13.00 (1.72)	12.89 (1.71)	13.20 (2.38)	14.44 (2.03)	*F* (3, 97) = 0.71 *p* = 0.55
Premorbid IQ (SD)	89.00 (10.84)	89.22 (14.89)	93.83 (13.48)	97.44 (11.87)	*F* (3, 97) = 1.38, *p* = 0.25
Sex (%)					χ^2^ (3) = 5.58, *p* = 0.13
Male	18 (69.2)	6 (33.3)	17 (56.7)	15 (55.6)	
Female	8 (30.8)	12 (66.7)	13 (43.3)	12 (44.4)	
Race/ethnicity (%)					χ^2^ (6) = 11.51, *p* = 0.07
Black/African American	23 (88.5%)	17 (94.4%)	20 (66.7%)	16 (59.3)	
White, non-Hispanic	1 (3.8%)	1 (5.6%)	3 (10.0%)	4 (14.8)	
Hispanic/Latino	2 (7.7%)	0 (0%)	7 (23.3%)	7 (25.9)	
Mood Symptoms (Current)					
Clinically Significant CES-D	11	5	9	5	χ^2^ (3) = 3.63, *p* = 0.30
Clinically Significant PSS	11	4	7	4	χ^2^ (3) = 5.63, *p* = 0.13
HIV Characteristics (SD)					
Years Living with HIV	16.58 (9.41)	16.13 (7.93)			t (38) = 0.16, *p* = 0.87
Current CD4 cell count	615.68 (272.18)	585.14 (242.64)			t (34) = 0.34, *p* = 0.74
HIV RNA Viral Load (copies/mL)	49.00 (2.69)	48.67 (3.94)			t (38) = 0.57, *p* = 0.75
Cocaine Dependence Characteristics					χ^2^ (2) = 0.573, *p* = 0.751
Cocaine Dependence within the past month (%)	11 (42.31%)		15 (51.72%)		
Cocaine Dependence within the past Year (%)	12 (46.15%)		13 (44.83%)		
Cocaine Dependence within the past 2 Years (%)	3 (11.54%)		2 (3.45%)		
Age first used cocaine (SD)	25.19 (8.66)		22.44 (5.43)		t (51) = 1.39, *p* = 0.17

Note: All values are means unless indicated otherwise. *N* = 101. HIV characteristics are based on 39 participants’ medical records out of the 44 individuals due to the inability to access medical records to confirm the self-reported lab values for the 5 participants.

**Table 2:** KMSK current and lifetime substance use scores for two cocaine-dependent groups.

M (SD)	Lifetime				Current			
	HIV+/CD+ (*n* = 26)	HIV−/CD+ (*n* = 30)	t	*p*	HIV+/CD+ (*n* = 26)	HIV−/CD+ (*n* = 30)	t	*p*
Alcohol	8.50 (3.57)	7.52 (3.70)	1.000	0.321	2.81 (2.83)	3.48 (2.65)	−0.910	0.367
Nicotine	8.58 (4.35)	7.93 (3.96)	0.573	0.569	5.69 (3.42)	4.69 (3.91)	1.015	0.315
Cocaine	19.38 (8.95)	15.52 (7.47)	1.75	0.9	4.46 (7.12)	5.14 (5.29)	−0.040	0.69
Methamphetamine	1.12 (3.26)	0.59 (2.97)	0.625	0.535	0.50 (1.77)	0.0 (0.0)	1.439	0.163
Heroin	0.69 (2.04)	0.38 (1.52)	0.640	0.525	0.0 (0.0)	0.03 (0.19)	−1.000	0.326
Cannabis	7.58 (5.99)	8.90 (5.80)	−0.828	0.412	1.04 (2.14)	2.59 (4.02)	−1.806	0.078

Note. *N =* 56. All values are means unless otherwise indicated. SD = Standard Deviation. KMSK = Kreek–McHugh–Schluger–Kellogg Scale. This table displays the KMSK lifetime and current values for the individuals in the two cocaine dependent groups (i.e., HIV+/CD+ and HIV−/CD+). Alcohol and Nicotine lifetime max score = 13, Cocaine lifetime max score = 32, Methamphetamine lifetime max score = 16, Heroin and Cannabis lifetime max score = 16. Alcohol current max score = 9, Nicotine current max score = 10, Cocaine current max score = 26, Methamphetamine current max score = 12, Heroin current max score = 10, Cannabis current max score = 13.

## Data Availability

The data are available from the corresponding author upon request.

## Supplementary Materials

The following supporting information can be downloaded at: https://www.mdpi.com/article/10.3390/v18010122/s1. Table S1: Neuropsychological Assessment Test Battery; Table S2: Raw Scores and Z-scores for each of the neurocognitive measures.
