# Supplementary material for: Cognitive Flexibility and Inhibition Deficits in HIV and Cocaine Dependence: Evidence from Stroop and Trail Making Tests
Source: Viruses. 2026 Jan 16;18(1):122. doi: 10.3390/v18010122 (PMC12846368; doi:10.3390/v18010122)
Supplement: Supplementary file 1 [file viruses-18-00122-s001.zip › viruses-4047188-supplementary.pdf]

Supplemental Table S1. Neuropsychological Assessment Test Battery.

Domain	Measure
Premorbid Intellectual Functioning	Wide Range Achievement Test-Revised: Word Reading (WRAT-W)
Attention / Executive Functions	Trail Making Test A and B<br><br>Conners' Continuous Performance Test, Third Edition<br>Stroop Color Word Test
Processing Speed	Symbol Digit Modalities Task (SDMT)
Learning and Memory	Hopkins Verbal Learning Test (HVLT)
Decision Making	Delay Discounting Task<br>Iowa Gambling Test

Supplemental Table S2. Raw Scores and Z-scores for each of the neurocognitive measures.

	Mean (SD)			
	HIV+/CD+ (*n* = 26)	HIV+/CD− (*n* = 18)	HIV−/CD+ (*n* = 30)	HIV−/CD− (*n* = 27)
Trail Making Test (SD)				
TMT-A Raw Score	34.27 (11.05)	35.44 (12.05)	29.97 (10.58)	30.74 (12.49)
TMT-A z-score	−0.16 (0.93)	−0.46 (0.76)	0.07 (0.98)	−0.17 (1.10)
TMT-A Errors	0.19 (0.40)	0.22 (0.43)	0.10 (0.31)	0.07 (0.27)
TMT-B Raw Score	101.04 (62.68)	121.22 (71.54)	107.43 (54.65)	106.86 (58.26)
TMT-B z-score	−0.04 (1.18)	−0.63 (1.34)	−0.50 (1.13)	−0.77 (1.31)
TMT-B Errors	1.04 (1.82)	1.22 (1.87)	1.33 (1.52)	1.44 (2.33)
TMT-B/A (Ratio)	2.90 (0.34)	3.22 (0.39)	3.85 (0.30)	3.66 (0.33)
Stroop Color Word Test (SD)				
Stroop Color Naming	71.45 (20.80)	68.05 (14.51)	67.78 (14.81)	70.00 (19.37)
Stroop Color Naming Errors	1.30 (3.08)	0.35 (0.71)	2.17 (4.07)	1.29 (1.58)
Stroop Word Reading	52.56 (14.42)	52.49 (10.32)	50.04 (10.53)	49.24 (11.77)
Stroop Word Reading Errors	1.00 (1.84)	0.27 (0.78)	0.87 (1.53)	0.41 (0.53)
Stroop Interference	167.90 (96.73)	138.85 (26.06)	130.45 (36.11)	115.08 (31.41)
Stroop Interference Errors	3.44 (5.82)	2.15 (3.54)	6.18 (6.81)	3.28 (4.37)
Stroop Int – Color Naming	100.31 (97.89)	71.11 (23.34)	62.37 (30.01)	41.82 (24.26)
Stroop Int – Word Reading	119.27 (99.00)	86.94 (24.98)	80.03 (33.53)	62.07 (22.88)

Note. *N* = 101. All values are means unless otherwise indicated. Raw scores for TMT-A, TMT-B, Stroop Color-Naming, Word-Reading, and Interference represent completion time in seconds.

SD = Standard Deviation.
